# *Chlamydia pneumoniae* Upsurge at Tertiary Hospital, Lausanne, Switzerland

**DOI:** 10.3201/eid3004.231610

**Published:** 2024-04

**Authors:** Florian Tagini, Onya Opota, Gilbert Greub

**Affiliations:** Institute of Microbiology, Lausanne University Hospital, Lausanne, Switzerland

**Keywords:** *Chlamydia pneumoniae*, outbreak, intracellular bacteria, asthma, community-acquired pneumonia, respiratory infections, pneumonia, bacteria, Switzerland

## Abstract

*Chlamydia pneumoniae* infection cases have usually accounted for <1.5% of community-acquired respiratory tract infections. Currently, Lausanne, Switzerland is experiencing a notable upsurge in cases, with 28 reported within a span of a few months. This upsurge in cases highlights the need for heightened awareness among clinicians.

The intracellular bacterium *Chlamydia pneumoniae* is a recognized cause of community-acquired pneumonia (*1*). High-frequency estimates were initially derived from serologic studies, but the advent of molecular techniques has revealed rates that are generally <1.5% among patients with respiratory tract infections, although epidemiological change between initial and current rate estimates cannot be ruled out (*2**,**3*). Sporadic outbreaks have been documented, such as a 2014 prison outbreak in Texas (*4*) and a 2016 community-acquired pneumonia outbreak in South Korea (*5*). In recent years, studies have also linked *C. pneumoniae* bacteria to bronchitis and asthma (*6*). *C. pneumoniae* bacteria has also been documented in patients with cystic fibrosis (*7*). Of note, infections occur at higher rates in children than in adults (*2*).

At the height of the SARS-CoV-2 pandemic, *C. pneumoniae* bacteria detection rates were low, paralleling the near-extinction state observed for *Mycoplasma pneumoniae* bacteria in Europe (*8*). However, a current rebound of *M. pneumoniae* infections is occurring (*9*). We report a similar increase in PCR-positive *C. pneumoniae* bacteria detection rates at a tertiary hospital in Switzerland. As the case series and the analysis thereof derive from the pathogen surveillance to which our institute is legally bound by the health authorities, Swiss legislation on human research is not applicable and the consent of the patients concerned is not required. This publication complies with the applicable data protection legislation and institutional guidelines.

During routine epidemiologic surveillance at Lausanne University Hospital in Lausanne, Switzerland, positive *C. pneumoniae* bacteria PCR rates surged to 3.61% during October–December 2023, peaking at 6.66% in October, contrasting with the usual 0%–0.75% range reported over the past decade (Figure 1, panel A, B). The PCR method we used for testing has been previously described in Opota et al. (*10*). In this most recent outbreak, we documented *C. pneumoniae* bacteria in 28 patients in 2023; of those, 20 were children (mean age 8 years) and 8 were adults (mean age 43 years). Patients with *C. pneumoniae* bacteria sometimes reported wheezing as a major clinical complaint. We tested bacterial loads in patients positive for *C. pneumoniae* bacteria and found that the mean bacterial load was 1,534,821 DNA copies/mL (range 200–11,998,897 DNA copies/mL). We collected nasopharyngeal swabs most frequently (n = 24), whereas we collected sputum samples (n = 5) and nasal swab samples (nostril only, n = 1) less frequently. Of note, bacterial loads were not higher in the analyzed sputa than in the nasopharyngeal swabs (p = 1 by Wilcoxon rank-sum test) (Figure 2). 

**Figure 1:**
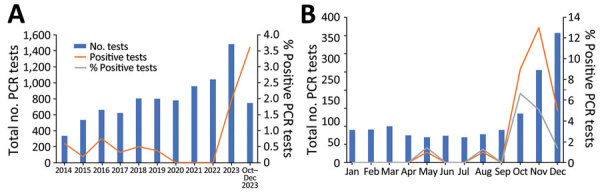
Positivity rate of *Chlamydia pneumoniae* PCRs in a tertiary care hospital, Lausanne, Switzerland. A) Yearly number of *C. pneumoniae* PCR tests conducted during 2014-2023. The final bar shows the last quarter of 2023, when the positivity rate exhibited a notable increase to 3.61%. B) Monthly numbers of *C. pneumoniae* PCR tests performed in 2023, showcasing positive tests and corresponding positivity rates. The data reveal a peak in the percentage of positivity of 6.66% in October.

**Figure 2:**
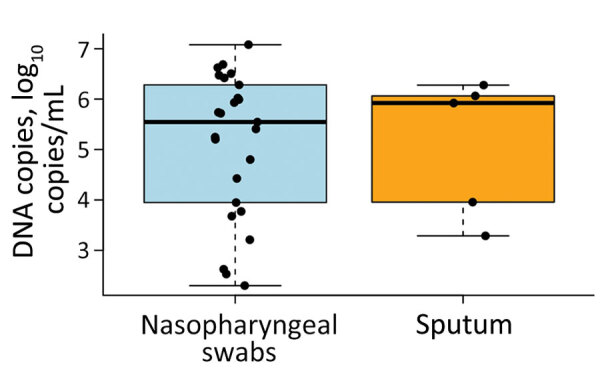
Boxplot of the quantifications of the *Chlamydia pneumoniae*–positive PCRs, by sample type, in a tertiary care hospital, Lausanne, Switzerland. In total, 24 nasopharyngeal swab and 5 sputum samples were available. For 2 patients, data were paired, with 1 nasopharyngeal swab and 1 sputum sample available for each. Black dots indicate individual samples; horizontal lines within boxes indicate medians; box tops and bottoms indicate interquartile range; and error bars indicate 1.5 times the value of the interquartile ranges. The nostril swab was omitted from the analysis. We observed no statistically significant difference between the groups (Wilcoxon rank-sum test).

The results of this analysis should be interpreted with caution in the absence of a larger number of paired samples. This analysis includes only 2 paired samples exhibiting <1 logarithm (decimal) of difference in DNA copies per milliliter. 

To explain this sudden surge of *C. pneumoniae* bacterial infection, we suspect 2 primary factors. First, decreased immunity may have developed because of fewer circulating strains in the population over the past 3 years, related to SARS-CoV-2 transmission prevention measures. Second, recently relaxed hygiene standards after the SARS-CoV-2 pandemic may have increased the risk for infection.

Clinical suspicion of *C. pneumoniae* infection is particularly warranted when patients’ clinical manifestations include a persistent dry cough or wheezing. Molecular testing, if available, should be the first-line diagnostic tool with nasopharyngeal swabs as an acceptable sample collection method. We do not recommend serologic testing in such cases because of the need to collect convalescent serum and the late appearance of antibodies. Antibodies generally develop 2–3 weeks after symptom onset for IgM and 4–8 weeks for IgG, which is rather late for diagnostic and therapeutic purposes. Furthermore, because *C. pneumoniae* bacterial infection can be treated by macrolides, doxycycline, or fluoroquinolones, current increases in both *C. pneumoniae* and *M. pneumoniae* bacteria lead us to recommend PCR testing for both bacteria in symptomatic patients, instead of testing only for respiratory viruses. Although co-infection with *M. pneumoniae* bacteria occurred in only 1 patient (*M. pneumoniae* PCR was tested on all samples) in our cohort, viral co-infections are not uncommon. Many multiplexed PCR respiratory panels are available and could help monitor the trend of *C. pneumoniae* bacterial infections on a larger scale.

In conclusion, we outline an upsurge of *C. pneumoniae* bacterial infections in the Lausanne region of Switzerland, especially in the pediatric population, raising concerns for other settings and regions. We found no clear epidemiologic link between patients, which suggests that we are detecting a minority of cases and that infections may occur at higher rates in the community than we have documented. This local finding highlights the importance of considering this intracellular bacterium as a causative agent, along with other fastidious organisms such as *M. pneumoniae* bacteria, which are also on the rise (*9*). 
